# Fungal-Infected Weeds: A Potential Source of Leaf Spot Disease in Rubber Trees from Southern Thailand

**DOI:** 10.3390/jof11030220

**Published:** 2025-03-14

**Authors:** Narit Thaochan, Chaninun Pornsuriya, Thanunchanok Chairin, Kodeeyah Thoawan, Putarak Chomnunti, Anurag Sunpapao

**Affiliations:** 1 Agricultural Innovation and Management Division (Pest Management), Faculty of Natural Resources, Prince of Songkla University, Hat Yai 90110, Thailand; narit.t@psu.ac.th (N.T.); chaninun.p@psu.ac.th (C.P.); thanunchanok.c@psu.ac.th (T.C.); kodeeyah.thoawan@gmail.com (K.T.); 2 School of Science, Mae Fah Luang University, Chiang Rai 507100, Thailand

**Keywords:** rubber tree, morphology, molecular techniques, weed

## Abstract

The rubber tree (*Hevea brasiliensis*) is an economically important crop in Thailand. Severe defoliation caused by emerging diseases has been reported to substantially reduce rubber yields during the leaf fall phase. The classical disease dispersal patterns of fungi in rubber tree plantations might be derived from weeds in adjacent fields. However, this hypothesis remains untested. Therefore, in this study, we collected and isolated fungi from symptomatic weed samples in rubber tree plantations in Krabi Province in southern Thailand. We found that *Parameria* sp. were dominant, showing the development of conidiomata on leaves. A total of 25 symptomatic *Parameria* sp. leaves were collected and tested for their pathogenicity on rubber tree leaves. The tests produced six fungal isolates, WC001, WC002, WL001, WL002, WN001, and WN002, that caused spots on the rubber tree leaves similar to those observed on the weeds. Morphological characterization revealed that fungal isolates WC001 and WC002 were *Colletotrichum* sp., WL001 and WL002 were *Lasiodiplodia* sp., and WN001 and WN002 were *Neopestalotiopsis* sp. Multigene phylogenetic analyses of combined *act*, *gapdh*, ITS, and *tub2* regions identified WC001 and WC002 as *Colletotrichum siamense*, while analyses of ITS, *tub2*, and *tef1-α* regions identified WL001 and WL002 as *Lasiodiplodia brasiliensis* and WN001 and WN002 as *Neopestalotiopsis cubana*. The occurrence of fungal diseases in rubber trees is significantly associated with leafy weeds in and around rubber tree plantations that could constitute reservoirs of fungal pathogens. The strategies used to control weeds have to be further considered in the future.

## 1. Introduction

The rubber tree (*Hevea brasiliensis*), belonging to family Euphorbiaceae, is an important flowering plant. It was originally located in the Amazon basin and is an economically important crop in the southwestern and northeastern regions of Thailand due to the milky latex it produces [1]. Rubber trees are mainly grown in Southeast Asia, especially Indonesia, Malaysia, Vietnam, and Thailand [2]. However, the climate change negatively impacts agricultural production, and altered temperature and precipitation patterns have been reported to shift pathogen distributions and increase disease incidence [3]. The rubber tree faces several diseases that can affect the natural rubber quality. Recent publications have reported increased incidences of fungal pathogen-associated diseases worldwide, particularly in tropical and subtropical regions. For instance, rubber tree leaf blight caused by *Neofusicoccum ribis* has been reported in Peninsular Malaysia [4], and rubber tree black spot disease caused by *Alternaria alternata* has been reported in China [5]. Indeed, leaf fall disease has been reported and linked to the fungus *Corynespora cassiicola*. This fungus is one of the most damaging illnesses and seriously impairs rubber tree plantations [6].

Pathogens affecting rubber trees employ diverse mechanisms that compromise tree health and productivity through systematic disruption of physiological processes. For instance, leaf pathogens such as *Corynespora cassiicola* and *Colletotrichum gloeosporioides* target the photosynthetic apparatus by penetrating leaf tissue and causing necrotic lesions. This reduces the tree’s photosynthetic capacity, severely limiting carbon assimilation and subsequent latex production [7]. Repeated defoliation events can deplete stored carbohydrate reserves, weakening trees and making them susceptible to secondary infections. Root pathogens, particularly *Rigidoporus microporus* causing white root disease and *Ganoderma philippii* causing red root disease, colonize and degrade woody root tissues through enzymatic breakdown of lignin and cellulose. This impairs water and nutrient uptake while destroying structural integrity [8]. As the infection progresses, the vascular system becomes compromised, often resulting in complete tree death within 6–12 months of symptom appearance. Panel diseases like bark necrosis and dry bark rot directly attack the latex vessel system. *Phytophthora palmivora* invades bark tissue through tapping wounds, causing localized tissue death and disrupting latex flow in affected areas. In severe cases, these infections can spread vertically along the trunk, rendering entire tapping panels unproductive for months or permanently [9]. Furthermore, many pathogens produce phytotoxins that trigger premature leaf senescence and inhibit cellular functions even in tissues not directly colonized. The *Pestalotiopsis* species, for example, produces toxins that interfere with stomatal regulation and membrane integrity, affecting tree-wide metabolic functions [10]. Climate variables significantly modulate disease progression, with high humidity and temperatures generally accelerating pathogen development cycles and increasing infection severity [11].

Powdery mildew caused by *Oidium heveae* and South American Leaf Blight (SALB) caused by *Microcyclus ulei*, though the latter is not yet present in Asia, represent significant threats to production [12]. These diseases can reduce latex yield and, in severe cases, cause complete plantation failure requiring costly replanting operations [13]. The economic impact extends beyond direct yield losses. Treatment costs, including fungicides and labor for application, add significantly to production expenses. A study by [14] estimated that disease management accounts for approximately 15–20% of total rubber plantation operational costs in Southeast Asia. Furthermore, the premature death of trees can reduce the economic lifespan of plantations from the expected 25–30 years to as few as 15 years, dramatically altering return-on-investment calculations for growers [15].

Thailand’s rubber industry, which employs over one million people and generates annual export revenues exceeding USD 6 billion, faces a potential economic loss due to pathogen-related issues [16]. These losses are expected to intensify with climate change, as altered precipitation patterns and increasing temperatures may favor pathogen proliferation and expand their geographical range [17]. The socioeconomic implications are particularly acute for smallholder farmers, who manage approximately 85% of Thailand’s rubber plantations. Without adequate resources for disease management, smallholders experience disproportionate economic hardship when outbreaks occur [18].

In Thailand, the total area of rubber tree plantations is approximately 3.2 million hectares, primarily in the tropical southern parts of Thailand. The weather in these areas with high temperatures (28–35 °C) and high relative humidity (>80%) favors pathogen germination and the spread of diseases. Recent studies have reported a new disease affecting rubber tree leaves in southern Thailand, associated with *Calonectria foliicola* [19], *Colletotrichum siamense* [20], *Lasiodiplodia chonburiensis*, and *L. theobromae* [21] as well as *Neopestalotiopsis cubana* and *N. formicarum* [22]. However, we found that some weeds in rubber plantations displayed leaf diseases like those observed in rubber trees. We hypothesized that some fungal pathogens infecting weeds in rubber tree plantations may act as inoculum sources that may cause diseases in rubber trees. Therefore, this research aims to isolate and identify fungal pathogens from weeds in rubber tree plantations and inoculate rubber tree leaves with the fungal pathogens to observe any incidence of disease.

## 2. Materials and Methods

### 2.1. Sample Collection and Fungal Isolation

A total of 25 symptomatic weed leaves from a rubber tree plantation were obtained from October to December 2021, during outbreaks of leaf fall and leaf spot diseases in Krabi Province, southern Thailand (7.909619, 99.163163). Infected samples were stored in plastic bags (20 × 20 cm) and then transported to the Plant Pathology Laboratory at the Faculty of Natural Resources, Prince of Songkla University, Hat Yai, Thailand. Fungi were isolated and characterized by a tissue transplanting method. Small pieces (0.5 × 0.5 cm) of healthy and infected tissues were cut, surface-disinfected in 0.5% sodium hypochlorite (NaClO) for 30 sec, and subsequently excess NaClO was removed with sterilized distilled water (DW) for 3 min twice. The samples were briefly dried on sterilized filter paper, then directly placed onto water agar (WA), and incubated at an ambient temperature (28 ± 2 °C) for three days. Hyphal tips were cut and transferred to potato dextrose agar (PDA) for morphological observations [23,24,25].

### 2.2. Morphology Study

Fungi were isolated from rubber tree leaves that showed disease symptoms, cultured on PDA, and incubated at an ambient temperature (28 ± 2 °C) with natural light to observe growth rates. Pure cultures were obtained after 2 cycles of purification. The mycelia were mounted on slides in water using a sterile needle. Their morphological characteristics were assessed with high-performance objectives under a Leica S8AP0 stereomicroscope (Leica Microsystems, Wetzlar, Germany) and a DM750 compound microscope (Leica Microsystems, Wetzlar, Germany).

### 2.3. Molecular Identification

The cultivated fungal isolates on PDA were incubated at an ambient temperature for 2 days to obtain the young mycelia. The extraction of DNA from fungal mycelia were assessed by a mini-preparation method [26]. We assessed the DNA quality via 1% agarose gel electrophoresis. PCR amplification was conducted using the BIO-RAD T100 Thermal Cycler (Bio-Rad Laboratories, Hercules, CA, USA). Specific regions of the internal transcribed spacer (ITS), β-tubulin 2 (tub2), and translation elongation factor 1-α (tef1-α) were amplified using the primer pairs ITS1/ITS4 [27], Bt2a/Bt2b [28], and EF1-728F/EF1-986R [29], respectively. They are designed for fungi in the genera *Lasiodiplodia* and *Neopestalotiopsis*. Portions of ITS, *tub2*, glyceraldehyde-3-phosphate dehydrogenase (*gapdh*), and actin (*act*) were, respectively, amplified using ITS5/ITS4 [27], Bt2a/Bt2b, GDF1/GDR1 [30], and ACT-512F/ACT-783R [29] primer pairs for the *Colletotrichum* genus. The PCR reaction mixture contained 2 μL DNA template, 20 pmol of each primer, 2 μL OneTaq^®^ PCR master mix with buffer (Biolabs, New England, MA, USA), and nuclease-free water. PCR amplification with an initial denaturation at 94 °C for 30 s was used, followed by 30 cycles of denaturation at 94 °C for 30 s, annealing at 55 °C for 60 s, extension at 72 °C for 1 min, and finally analyzed with a final extension at 72 °C for 5 min [31]. The stained portions of the PCR products were performed using Novel Juice (GeneDirect, Taoyuan, Taiwan) and observed through 1% agarose gel electrophoresis. We used the Macrogen Sequencing Service (Macrogen, Seoul, Republic of Korea), utilizing the same primers employed during the PCR amplification to detect the sequencing of PCR products. The DNA sequences of the fungal isolates in this study were subjected to a Blastn search to compare them with known sequences in the NCBI (National Center for Biotechnology Information) database. DNA sequences were aligned using MEGA version 10 [32]. The generated sequences in this study were aligned with the known sequences in the NCBI (National Center for Biotechnology Information) database using MAFFT v. 7.0 online servers (http://mafft.cbrc.jp/alignment/server/index.html, accessed on 2 March 2024) and manually adjusted as necessary in MEGA X. Phylogenetic analysis of the combined ITS, tub2, and tef1-α DNA sequences for *Lasiodiplodia* and *Neopestalotiopsis*, as well as the combined act, gapdh, ITS, and tub2 sequences for *Colletotrichum*, was performed employing the maximum likelihood (ML), maximum parsimony (MP), and Bayesian inference (BI). Maximum likelihood trees were constructed using MEGA X based on the K2 + G evolution model. Maximum parsimonious trees were obtained using the heuristic search option with 1000 random additions of sequences and tree bisection and reconnection (TBR) as the branch-swapping algorithm of MEGA X. The Bayesian tree was generated using MrBayes ver. 3.2.7. Two parallel Markov chain Monte Carlo (MCMC) runs were performed for 1,000,000 generations and were sampled every 100 generations. The initial 1000 generations were discarded as burn-in, and the remaining trees were used to calculate the Bayesian inference posterior probability (BPP) values. The phylogenetic trees were visualized using FigTree v1.4.0 (http://tree.bio.ed.ac.uk/software/figtree/, accessed on 8 March 2024).

### 2.4. Pathogenicity Test

To determine whether fungi isolated from *Parameria* sp. could cause disease on rubber tree leaves, a pathogenicity test by agar plug with fungal mycelia was conducted on healthy leaves. Fungal isolations were performed and cultured on PDA for 7 days at ambient temperature and natural light (12:12 h). Rubber tree leaves (RRIM600) were pierced with fine needles, and agar plugs containing fungal mycelia were directly placed on the wounds. Agar plugs without fungal mycelia were used as the control. The experiment was carried out in three replicates and was repeated twice. Inoculated leaves were kept humid in a moist box (90–95% RH), incubated at 28 ± 2 °C with natural light, and lesion development was observed daily.

### 2.5. Use of AI-Assisted Tools in Manuscript Preparation

During the early stages of manuscript preparation, ChatGPT-4o (OpenAI) was used to assist in generating background ideas and improving language clarity. The AI tool was not used for data analysis, interpretation of results, or experimental design. All scientific data, analyses, interpretations, and final decisions were validated and verified by the authors.

## 3. Results

### 3.1. Symptom Recognition

**Figure 1:**
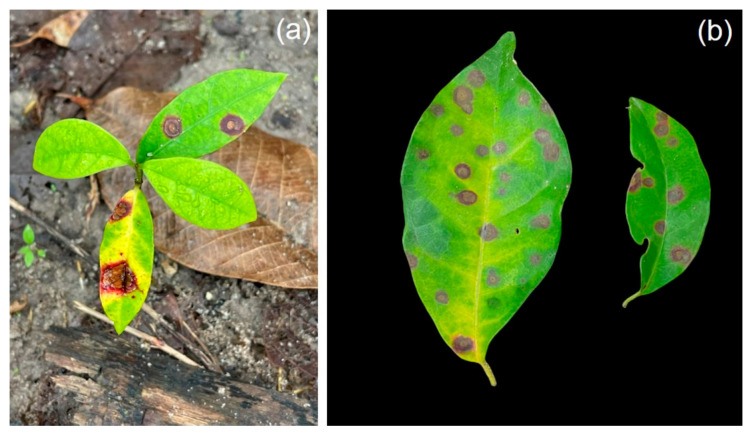
Symptomatic leaf samples from the weed *Parameria* sp.: brown lesions expanded, forming dark brown margins (**a**) and dark brown to green lesions (**b**).

The leaves of infected *Parameria* sp. presented similar features to the classic features of infected rubber tree leaves (Figure 1). Spots were initially uniform, small, and circular, light brown to dark brown in color, and later expanded, forming dark irregular margins (Figure 1). Several formed irregular branched pycnidia and some old lesions showed dense pycnidia.

### 3.2. Morphology Study

**Figure 2:**
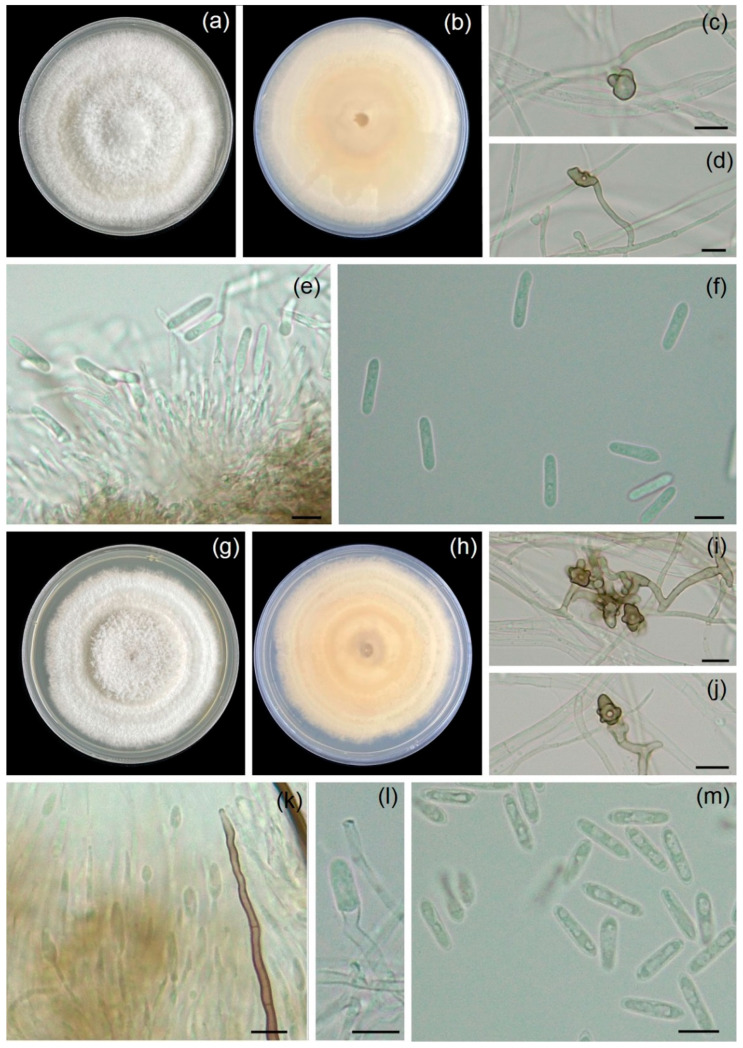
Microphotographs showing the morphological characteristics of *Colletotrichum* sp. including WC001 and WC002: top view of colony on PDA (**a**,**g**); bottom view of colony on PDA (**b**,**h**); appressoria (**c**,**d**,**i**,**j**); conidiophores and conidia (**e**,**k**,**l**); and conidia (**f**,**m**). Bars (**c**–**f**,**i**–**m**) = 10 µm.

The six fungal isolates were classified from morphological characteristics into three fungal genera, namely *Colletotrichum* (WC001 and WC002), *Lasiodiplodia* (WL001 and WL002), and *Neopestalotiopsis* (WN001 and WN002). The occurrence of fungal isolates (WC001 and WC002) in PDA covered an area of 7.8 cm in the Petri dish within 7 days, demonstrating a growth rate of 11.1 mm/day. Colonies exhibited a cottony material, pale, or white–gray color (Figure 2). The conidiomata presented a sticky pink to orange coloration and was darker in the center of the dish. The conidiophores were elongated and hyaline with openings. The conidia were cylindrical with round ends, non-septate and smooth, and 5.1–7.9 (6.4 ± 0.8) μm wide and 19.2–27.9 (24.6 ± 2.6) μm long ( $\bar{x}$ = 6.4 × 24.6 μm, n = 20). Based on their morphologies, fungal isolates WC001 and WC002 were identified as *Colletotrichum* sp. [23].

**Figure 3:**
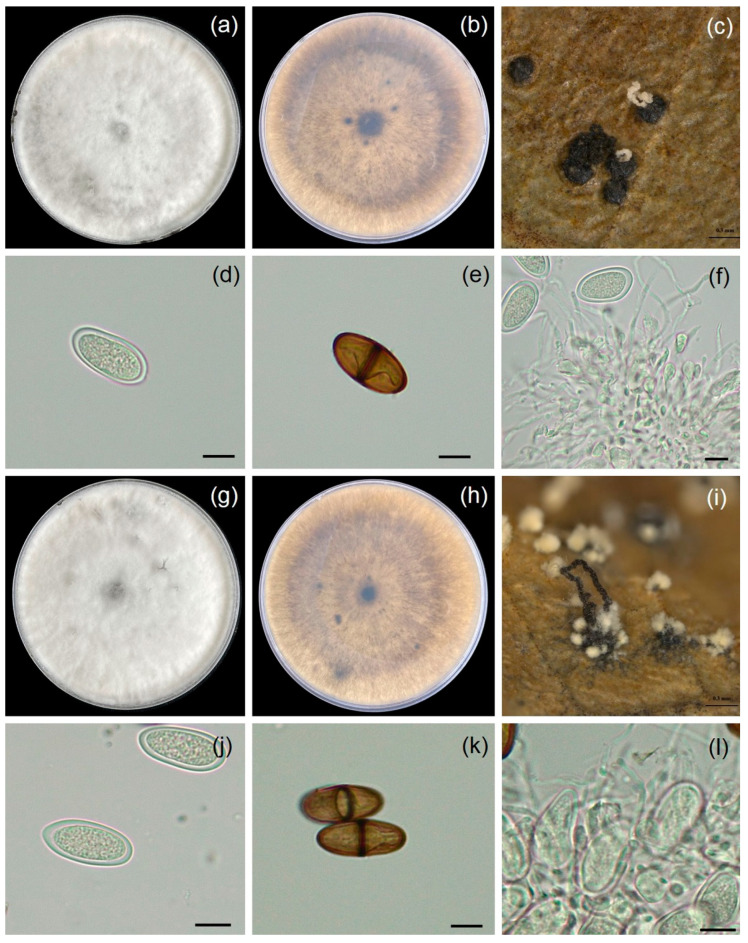
Microphotographs showing the morphological characteristics of *Lasiodiplodia* sp. (WL001 and WL002): top view of colony on PDA (**a**,**g**); bottom view of colony on PDA (**b**,**h**); pycnidia (**c**,**i**); immature conidia (**d**,**j**); mature conidia (**e**,**k**); and conidiogenous cells and paraphyses (**f**,**l**). Bars (**d**–**f**,**i**–**l**) = 10 µm.

Fungal isolates WL001 and WL002 were grown in PDA, covering 8.2 cm of the Petri dish within 7 days, indicating a growth rate of 11.7 mm/day at ambient temperature. Colonies initially appeared as white to pale greenish gray and changed into a dark with age (Figure 3). Pycnidia developed on leaf surfaces. They were solitary or aggregate, globose to subglobose to irregular in shape, and brown to dark brown in color, depending on the isolate. Paraphyses were hyaline and septate. Both immature and mature conidia were exhibited as sub-ovoid to ellipsoid with round apices. Immature conidia were aseptate, double-layered, hyaline, unicellular, and 11.8–14.2 μm. Mature conidia were dark brown, septate, 24.1–29.9 (27.8 ± 1.8) μm long, and 13.4–15.6 (14.5 ± 0.8) μm wide ( $\bar{x}$ = 27.8 × 14.5 μm, n = 20). Hence, the fungal isolates (WL001 and WL002) were classified as *Lasiodiplodia* sp. based on morphological characteristics [24].

**Figure 4:**
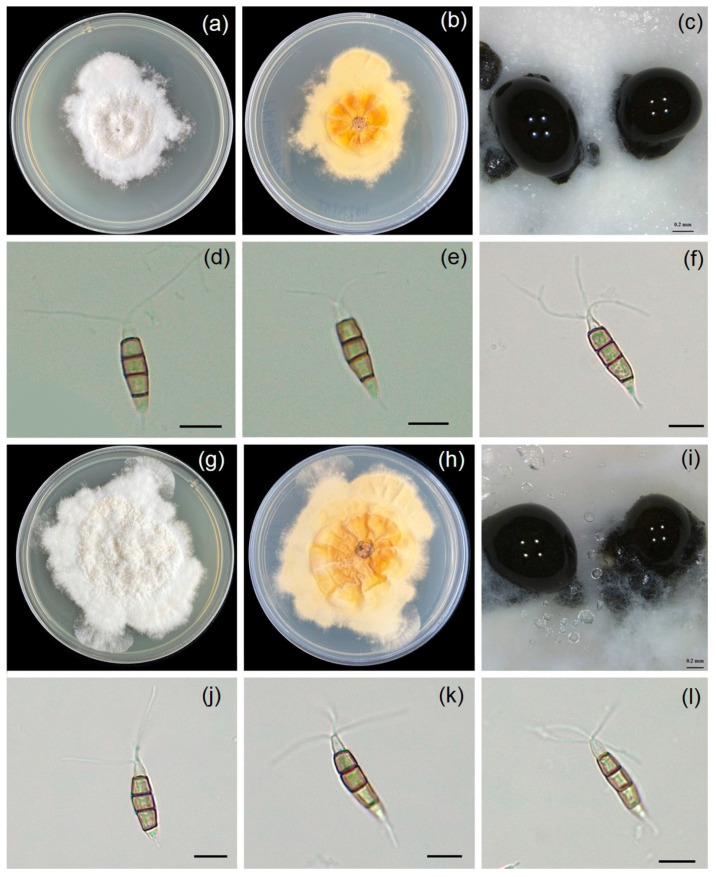
Microphotographs showing the morphological characteristics of *Neopestalotiopsis* sp. isolates WN001 and WN002: top view of colony on PDA (**a**,**g**); bottom view of colony on PDA (**b**,**h**); conidiomata with black slimy exudate (**c**,**i**); and conidia with versicolored median cells (**d**–**f**,**j**–**l**). Bars (**d**–**f**,**j**–**l**) = 10 µm.

Fungal isolates WN001 and WN002 were grown in PDA and covered 6.5 cm of the Petri dish within 7 days, indicating a growth rate of 9.3 mm/day at ambient temperature. Colonies were white to pale brown on PDA with lobate edges, and conidiomata presented as black slimy conidial masses embedded in mycelia (Figure 4). Conidia were fusoid to ellipsoid, slightly curved, 20.5–31.4 (24.9 ± 3.1) μm long, and 5.1–6.7 (6.1 ± 0.5) μm wide ( $\bar{x}$ = 24.9 × 6.1 μm, n = 20), comprising five cells. The three median cells were versicolored, apical cells were conical, and hyaline with two to three apical appendages 12.1–27.9 (20.8 ± 3.7) μm long. The basal cells were conical hyaline with one basal appendage 4.2–6.8 (4.9 ± 0.9) μm ( $\bar{x}$ = 20.8 × 4.9 μm, n = 30) long. According to our morphological characteristics, it was noted that the fungal isolates (WN001 and WN002) were identified as *Neopestalotiopsis* sp. [25].

As mentioned above, there are six fungal isolates stored in the culture collection of Pest Management, Faculty of Natural Resources, Prince of Songkla University, Thailand, under the accession numbers PSU-WC001, PSU-WC002, PSU-WL001, PSU-WL002, PSU-WN001, and PSU-WN002.

### 3.3. Molecular Identification

**Figure 5:**
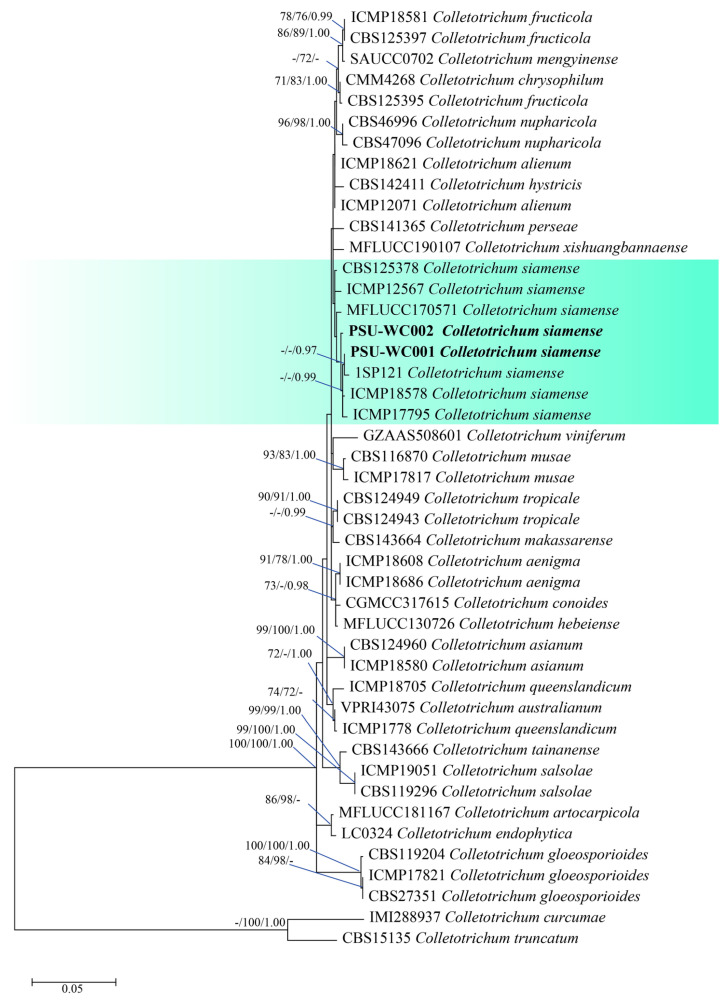
The phylogenetic tree with the combined ITS, *gapdh, act*, and *tub2* sequences. The phylogenetic relationships between *Colletotrichum* species in the *Colletotrichum gloeosporioides* species complex. Bootstrap support values above 70% and Bayesian posterior values above 0.95 are shown at each node (ML/MP/PP). *Colletotrichum truncatum* CBS 15135 and *Colletotrichum curcumae* IMI 288937 were used as outgroups. The green background indicates *C. siamense* and strains generated in this study are indicated in bold.

To identify the six fungal isolates at the species level, the ITS, *tub2*, and *tef1-α* portions of PSU-WL001, PSU-WL002, PSU-WN001, and PSU-WN002 were sequenced, and the *act*, *gapdh*, ITS, and *tub2* portions of PSU-WC001 and PSU-WC002 were sequenced. The Blastn search results for PSU-WC001 and PSU-WC002 warranted the genus *Colletotrichum*. A phylogenetic tree was composed of two isolates and 43 reference sequences from *Colletotrichum* sp. *C. curcumae* and *C. truncatum*, while the tree with single loci and the concatenated data set (*act*, *gapdh*, ITS, and *tub2*) were demonstrated. The alignment used 217 bases for *atc*, 238 bases for *gapdh*, 448 bases for ITS, and 366 bases for *tub2*. PSU-WC001 and PSU-WC002 were grouped together with *C. siamense* (Figure 5). The partial DNA sequences of *act*, *gapdh*, ITS, and *tub2* were submitted in GenBank via the accession numbers (Table S1).

**Figure 6:**
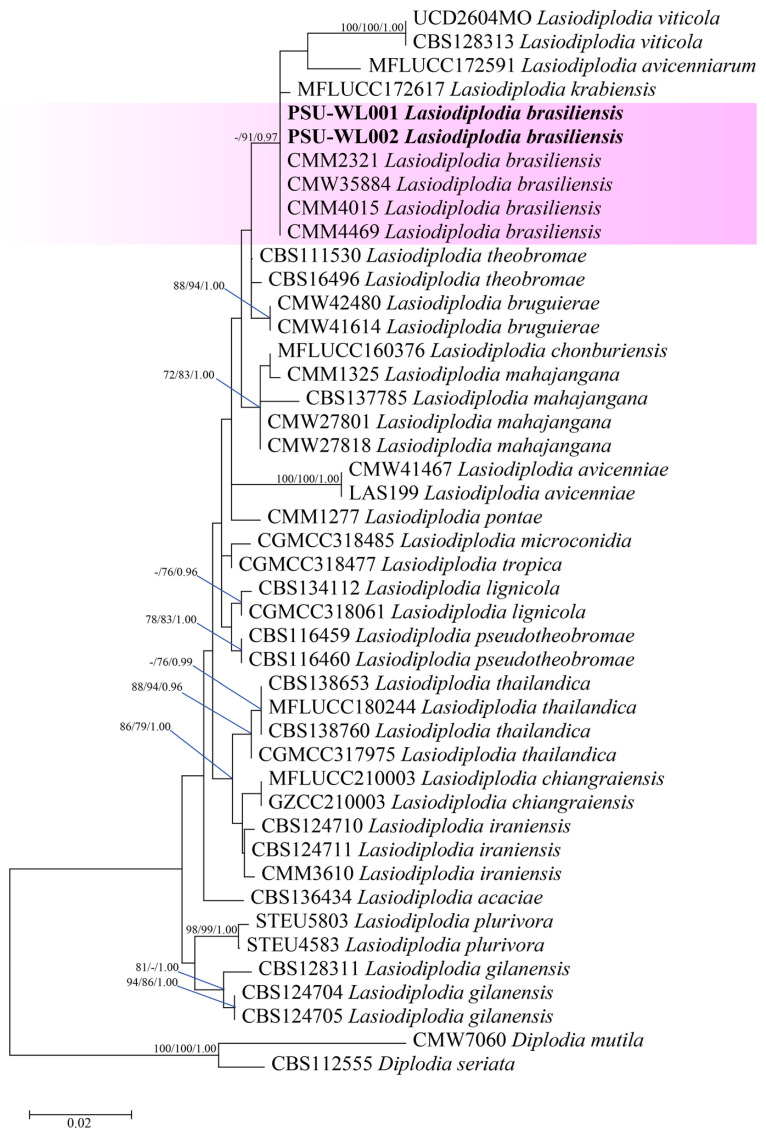
The phylogenetic tree with combined ITS, *tef1-α*, and *tub2* sequences. The phylogenetic relationships between *Lasiodiplodia* species. Bootstrap support values above 70% and Bayesian posterior values above 0.95 are shown at each node (ML/MP/PP). *Diplodia mutila* CMW7060 and *Diplodia seriata* CBS112555 were used as outgroups. The purple background indicates *Lasiodiplodia brasiliensis* and strains generated in this study are indicated in bold.

The Blastn search of PSU-WL001 and PSU-WL002 showed they belonged to the genus *Lasidiplodia* and had a 99% sequence similarity to *L. brasiliensis*. The phylogenetic tree between the combined DNA sequences of ITS (487 bases), *tub2* (379 bases), and *tef1-α* (280 bases) and the sequences of two isolates was aligned with those of 41 references of *Lasiodiplodia* and two outgroups (*Diplodia mutila* and *D. seriata*). The analysis of maximum likelihood showed that the phylogenetic placement of two isolates was closely associated with an isolate of *L. brasilensis* (Figure 6). It highlights the partial DNA sequences for ITS, tub2, and tef1-α, which are submitted to GenBank, with the corresponding accession numbers listed in Table S2.

**Figure 7:**
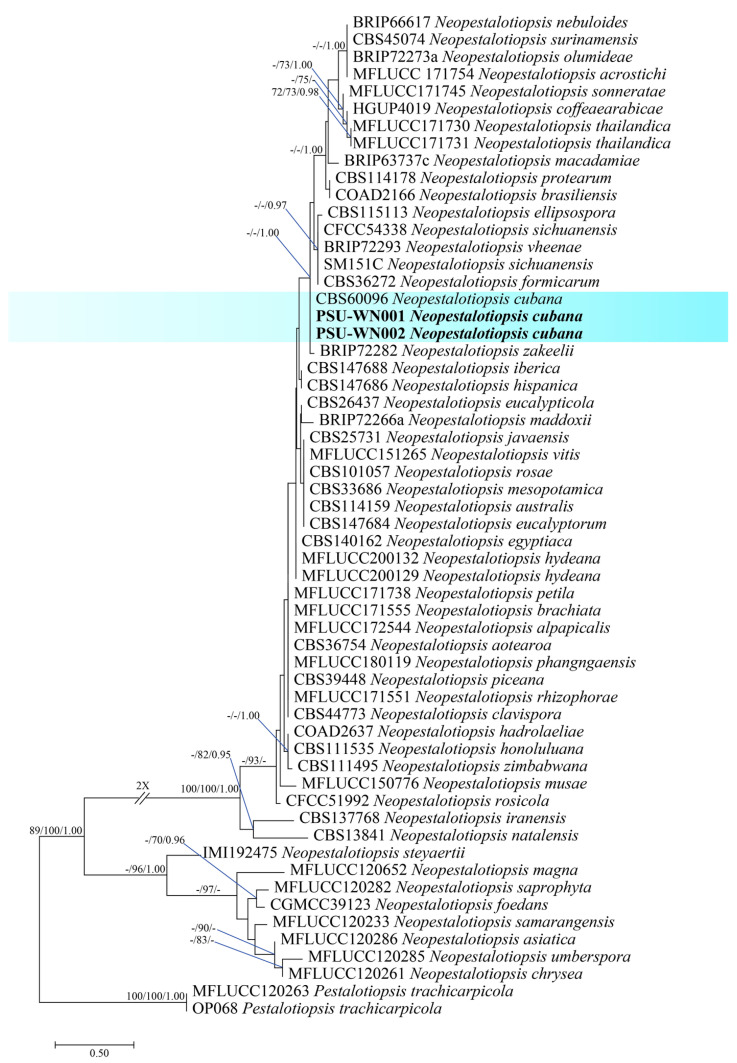
The phylogenetic tree was generated by maximum likelihood analysis based on combined ITS, *tef1-α*, and *tub2* sequences. The phylogenetic relationships between *Neopestalotiopsis* species. Bootstrap support values above 70% and Bayesian posterior values above 0.95 are shown at each node (ML/MP/PP). *Pestalotiopsis trachicarpicola* CMW7060 and OP068 were used as outgroups. The blue background indicates *Neopestalotiopsis cubana* and strains generated in this study are indicated in bold.

The Blastn search of PSU-WN001 and PSU-WN002 revealed they belonged to the genus *Neopestalotiopsis*. The alignment composed of 427 bases for ITS, 390 bases for *tub2*, and 476 bases for *tef1-α*. The sequences of the two isolates obtained in the present study were aligned with the sequences of *Neopestalotiopsis* and an outgroup (*Pestalotiopsis trachycarpicola*). PSU-WN001 and PSU-WN002 grouped together with *N. cubana* (Figure 7). The partial DNA sequences of *act*, *gapdh*, ITS, and *tub2* are submitted in GenBank with the accession numbers in Table S3.

### 3.4. Pathogenicity Test

**Figure 8:**
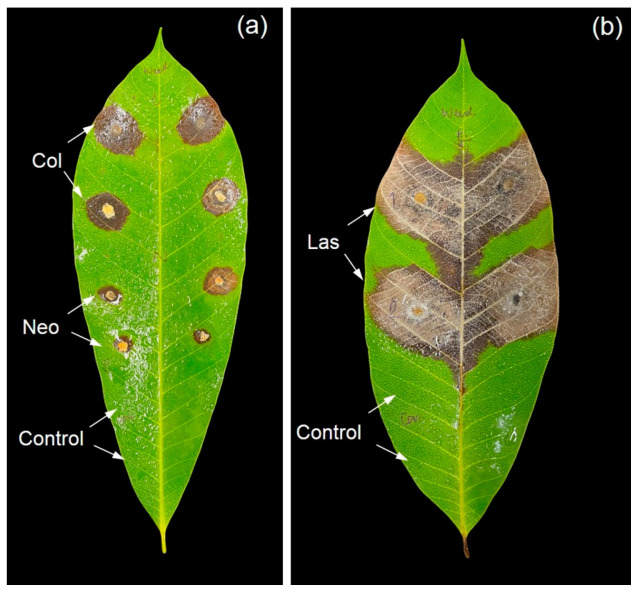
Pathogenicity test of fungal isolates on healthy rubber leaves: leaves were inoculated with *Colletotrichum* sp. (Col, WC001 [upper] and WC002 [lower]), *Neopestalotiopsis* sp. (Neo, WN001 [upper] and WN002 [lower]), control uninoculated, (**a**) and with *Lasiodiplodia* sp. (WL001 [upper] and WL002 [lower]) and control (**b**).

After 7 days, the symptoms and signs of leaf disease began to appear on the inoculated rubber tree leaves (Figure 8). The fungal pathogens were re-isolated from the inoculated plants to observe their morphological examinations. The pathogenic morphology remained consistent with the characteristics noted before entering the inoculation process.

## 4. Discussion

Fungal pathogens were isolated from weeds (*Parameria* sp.) found in rubber tree plantations in southern Thailand. Six isolates (PSU-WC001, PSU-WC002, PSU-WL001, PSU-WL002, PSU-WN001, and PSU-WN002) effectively infected rubber tree leaves that showed leaf spot and leaf blight symptoms similar to those observed in previous studies [19,20,21,22]. The pathogens were identified from morphological characteristics and molecular alignments of multiple DNA sequences as *C. siamense* (PSU-WC001 and PSU-WC002), *L. brasiliensis* (PSU-WL001 and PSU-WL002), and *N. cubana* (PSU-WN001 and PSU-WN002). The results of both characterization methods were in agreement with previous reports that the information of morphology and molecular studies is to successfully identify fungal pathogens.

*Colletotrichum siamense* belongs to the *C. gloeosporioides* species complex and caused diseases in a number of plant species, including anthracnose in avocado [33], anthracnose in papaya [34], and leaf blotch in *Viburnum odoratissimum* [35]. *Lasiodiplodia brasiliensis* has been documented to cause root rot in watermelon [36] and root rot in melon plants [37]. *N. cubana* has been reported to cause leaf blight in *Ixora chinensis* [38] and leaf fall in *H. brasiliensis* [22]. Although both *C. siamense* and *N. cubana* have been previously reported to cause leaf spot and leaf fall disease in southern parts of Thailand, our findings showed *L. brasilensis*, for the first time, in weeds in the region and that it was able to cause leaf spot disease on rubber leaves. However, certain phytopathogenic fungi are able to infect some weeds and can be used as biological weed control agents. For instance, *C. graminicola* and *Exserohilum fusiforme* have been used to control *Echinochloa crus-galli* and *Echinochloa* spp. [39], and the application of *Alternaria* sp. has successfully controlled *Rumex dentatus* [40].

This study found that *N. cubana* from the weed *Parameria* sp. can cause disease in rubber trees, which is consistent with the previous report by Pornsuriya et al. [22], who identified this fungus as the cause of rubber leaf fall disease in southern Thailand. However, there have been no reports of *N. cubana* infecting rubber trees in other Southeast Asian countries, such as Malaysia, Indonesia, or Vietnam. Despite this, other species of *Neopestalotiopsis* have been reported to cause damage to rubber trees, such as *N. clavispora* in Malaysia [41] and *N. rosae* in China [42]. This indicates that fungi in the genus *Neopestalotiopsis* play a significant role in rubber tree diseases in this region.

In southern parts of Thailand, increased incidences of a number of diseases caused by fungi in the phylum Ascomycota have been recently reported in several plant species. In this study, three fungal pathogens (*C. siamense*, *L. brasiliensis*, and *N. cunbana*) in the phylum Ascomycota were isolated from *Parameria* sp., which are weeds commonly found in rubber plantations. Recently, leaf fall and leaf spot have been observed in rubber trees in these areas too [19,20,21,22]. It is possible that some weeds in rubber tree plantations may be a source of plant pathogenic fungi that infect the trees and may be associated with leaf disease in rubber trees.

To fulfill Koch’s postulates, the same pathogen should be inoculated onto the same host species by pathogenicity test. However, we did not directly inoculate pathogens found in this study onto weeds because we could not prepare healthy weed plants (no commercial seed available) for inoculation. We strongly wanted to observe whether fungi in weeds could cause leaf disease on rubber leaves. Therefore, we substituted inoculated fungal pathogens isolated from weeds to rubber tree leaves. Although weeds are considered a significant plant pest in crop production, their environmentally friendly management is crucial for agriculture to avoid yield losses. Our findings revealed at least three fungal species that could infect weeds and cause diseases, which may be useful for the biological control of weeds in rubber tree plantations. However, at least two of the fungal pathogens identified in this study, *C. siamense* and *N. cubana*, have been reported to cause leaf spot disease and leaf fall disease, respectively. Our results revealed that fungal strains isolated from weeds can infect rubber trees and cause leaf disease. This basic knowledge can prove useful in future surveillance and disease management strategies.

In southern Thailand, *Colletotrichum siamense*, *Lasiodiplodia brasiliensis*, and *Neopestalotiopsis cubana* have been identified as key fungal pathogens affecting rubber tree plantations. The infection process of these fungi likely follows a cycle involving alternative hosts, such as weeds, and environmental factors conducive to disease spread. *C. siamense* belongs to the *C. gloeosporioides* species complex and has been reported to cause anthracnose in multiple plant species, including rubber trees [20]. The pathogen spreads through conidia dispersed by rain splash and wind, allowing rapid colonization of host plants. *L. brasiliensis*, known to cause root rot in melon plants [37], was first identified in weeds within rubber plantations in this study, indicating its potential role as a latent pathogen in the ecosystem. This fungus produces pycnidia containing spores that can be disseminated by insects or rain, leading to infections in rubber trees. *N. cubana*, previously reported as the causal agent of leaf fall disease in rubber trees in Thailand [22], exhibits a similar infection pattern. The fungus produces multicellular conidia that attach to leaf surfaces, penetrate through stomata or wounds, and cause necrotic lesions. While *N. cubana* has been confirmed in Thailand, it has not yet been reported in other Southeast Asian rubber plantations, such as in Malaysia or Vietnam. However, closely related species like *N. rosae* have been associated with rubber tree diseases in China [42]. The presence of these fungi on both rubber trees and surrounding weeds suggests a potential reservoir for pathogen survival and transmission. Future studies should explore whether infected weeds serve as primary inoculum sources and investigate integrated disease management strategies to mitigate fungal spread in rubber plantations.

## Data Availability

The original contributions presented in this study are included in the article/Supplementary Materials. Further inquiries can be directed to the corresponding authors.

## Supplementary Materials

The following supporting information can be downloaded at https://www.mdpi.com/article/10.3390/jof11030220/s1: Table S1: Collection details and GenBank accession numbers of *Colletotrichum* isolates in this study; Table S2: Collection details and GenBank accession numbers of *Lasiodiplodia* isolates in this study; and Table S3: Collection details and GenBank accession numbers of *Neopestalotiopsis* isolates in this study.
