# Correction: Thaochan et al. Fungal-Infected Weeds: A Potential Source of Leaf Spot Disease in Rubber Trees from Southern Thailand. *J. Fungi* 2025, *11*, 220

**DOI:** 10.3390/jof12060406

**Published:** 2026-06-03

**Authors:** Narit Thaochan, Chaninun Pornsuriya, Thanunchanok Chairin, Kodeeyah Thoawan, Putarak Chomnunti, Anurag Sunpapao

**Affiliations:** 1Agricultural Innovation and Management Division (Pest Management), Faculty of Natural Resources, Prince of Songkla University, Hat Yai 90110, Thailand; narit.t@psu.ac.th (N.T.); chaninun.p@psu.ac.th (C.P.); thanunchanok.c@psu.ac.th (T.C.); kodeeyah.thoawan@gmail.com (K.T.); 2School of Science, Mae Fah Luang University, Chiang Rai 507100, Thailand

## Text Correction

There was an error in the original publication regarding several quantitative statements in the Introduction section [1]. Some numerical estimates were not sufficiently supported by directly verifiable references and may have resulted in overstatement or misinterpretation. To address this issue, the numerical values “by 20–45%” (in paragraph 2), “>85%” (in paragraph 2), “between 25–30 °C” (in paragraph 2), “by 30–40% annually” (in paragraph 3), and “of $450–700 million annually” (in paragraph 4) have been removed, and the text has been revised to present the information in a more general and accurate manner. The scientific conclusions are unaffected.

A new section should be added to 2.5. Use of AI-Assisted Tools in Manuscript Preparation.

2.5. Use of AI-Assisted Tools in Manuscript Preparation

During the early stages of manuscript preparation, ChatGPT-4o (OpenAI) was used to assist in generating background ideas and improving language clarity. The AI tool was not used for data analysis, interpretation of results, or experimental design. All scientific data, analyses, interpretations, and final decisions were validated and verified by the authors.

## References

ChatGPT was used at an early drafting stage to generate background ideas and references, and some of these references were inaccurate or non-existent and were not sufficiently verified before submission. These incorrect references subsequently remained in the submitted, revised, and published versions. Replacing original references [6,7,8,9,10,11,12,13,14,15,16,17,18,39,41,42]:

6. Pu, J.; Zhang, X.; Qi, Y.; Xie, Y.; Zhang, H.; Zhang, H. First record of Corynespora leaf fall disease of Hevea rubber tree in China. *Australas. Plant Dis. Notes*
**2007**, *2*, 35–36.

7. Hashim, I.; Chee, K.H.; Duncan, E.J. Damage assessment and yield losses due to Corynespora leaf fall disease in mature rubber plantations. *J. Rubber Res.*
**2019**, *22*, 217–226.

8. Ogbebor, N.O.; Omorusi, V.I.; Orumwense, K. Biochemical mechanisms of rubber tree root degradation by *Rigidoporus microporus* and implications for control strategies. *For. Pathol.*
**2022**, *52*, e12715.

9. Sreelatha, S.; Joseph, K. Anatomical and biochemical alterations in rubber tree bark following Phytophthora infection and their relation to latex yield reduction. *Trees*
**2021**, *35*, 145–157.

10. Wong, M.Y.; Zulkifli, Y.; Sunderasan, E. Phytotoxins produced by Pestalotiopsis species in rubber plantations: Detection, characterization and significance. *Eur. J. Plant Pathol.*
**2020**, *156*, 1151–1163.

11. Liyanage, K.K.; Khan, S.; Brooks, S. Climate-dependent development of rubber tree pathogens: Implications for disease management under environmental change. *Plant Pathol.*
**2018**, *67*, 1026–1038.

12. Liyanage, A.S.; Peries, O.S.; Liyanage, N.I.S. Major diseases of rubber and their management. In *Natural Rubber: Biology, Cultivation and Technology*; Elsevier Science Publishers: Amsterdam, The Netherlands, 2016; pp. 213–238.

13. Jayasinghe, C.K. Rubber tree diseases: Economic impact and management strategies. *Mycosphere*
**2020**, *11*, 795–817.

14. Norhayati, M.; Nair, H.K.M.; Wong, M.Y. Economic assessment of fungicide application in rubber plantations in Malaysia. *J. Plant. Crops*
**2019**, *47*, 182–191.

15. Vongkhamheng, C.; Phimmavong, S.; Koutrakis, E. Economic impacts of white root disease on smallholder rubber plantations in Northern Laos. *Forests*
**2022**, *13*, 512–526.

16. European Forest Institute and Rubber Authority of Thailand. Briefing—Thailand’s Natural Rubber Producers Are Preparing for New Market Requirements. Available online: https://efi.int/sites/default/files/files/publication-bank/2024/Briefing%20-%20Thailand’s%20natural%20rubber%20producers%20are%20preparing%20for%20new%20market%20requirements.pdf (accessed on 28 February 2025).

17. Puengcharoen, S.; Sirisomboon, P.; Duangpatra, J. Climate change effects on rubber tree diseases in Thailand: A review and future perspectives. *Plant Pathol.*
**2021**, *70*, 1265–1280.

18. Chambon, B.; Ruf, F.; Kongmanee, C.; Angthong, S. Can the cocoa industry solve the rubber smallholders’ crisis? A proposal from Thailand. *For. Policy Econ.*
**2018**, *85*, 132–141.

39. Johanson, D.; Wyse, D.; Janes, K. Controlling weeds phytopathogen. *Weed Tech.*
**2003**, *10*, 621–624.

41. Ngobisa, A.I.C.N.; Zainal Abidin, M.A.; Wong, M.Y.; Noordin, W.D. Characterisation and pathogenicity of Pestalotiopsis species associated with leaf disease of rubber (*Hevea brasiliensis*). *Arch. Phytopathol. Plant Protect.*
**2015**, *48*, 448–457.

42. Zhao, F.; Wang, D.; Tao, Z.; Fan, Z.; Guo, H.; Zhang, H.; Huang, L. First report of Neopestalotiopsis rosae causing leaf spot of rubber tree in China. *Plant Dis.*
**2019**, *103*, 2947.

After correction, the References [6,7,8,9,10,11,12,13,14,15,16,17,18,39,41,42] became as follows:6.Jinji, P.; Xin, Z.; Yixian, X.; Huiqiang, Z.; He, Z. First record of Corynespora leaf fall disease of Hevea rubber tree in China. *Australas. Plant Dis. Notes*
**2007**, *2*, 35–36.7.Manju, M.J.; Benagi, V.I.; Shankarappa, T.H.; Jacob, C.K.; Vinod, K.K. Dynamics of Corynespora leaf fall and Colletotrichum leaf spot diseases of rubber plants (*Hevea brasiliensis*). *J. Mycol. Plant. Pathol.*
**2014**, *44*, 108.8.Saidi, N.B.; Al-Obaidi, J.R.; Fisol, A.F.B.C. *Rigidoporus microporus* and the white root rot disease of rubber. *For. Pathol.*
**2023**, *53*, e12794.9.Carella, P.; Gogleva, A.; Tomaselli, M.; Alfs, C.; Schornack, S. *Phytophthora palmivora* establishes tissue-specific intracellular infection structures in the earliest divergent land plant lineage. *Proc. Natl. Acad. Sci. USA*
**2018**, *115*, E3846–E3855.10.Wu, C.; Wang, Y.; Yang, Y. *Pestalotiopsis* Diversity: Species, Dispositions, Secondary Metabolites, and Bioactivities. *Molecules*
**2022**, *27*, 8088.11.Velásquez, A.C.; Castroverde, C.D.M.; He, S.Y. Plant–pathogen warfare under changing climate conditions. *Curr. Biol.*
**2018**, *28*, R619–R634.12.Guyot, J.; Le Guen, V. A review of a century of studies on south american leaf blight of the rubber tree. *Plant Dis.*
**2018**, *102*, 1052–1065.13.Chen, L.; Xu, L.; Li, X.; Wang, Y.; Feng, Y.; Huang, G. The diseases and pests of rubber tree and their natural control potential: A bibliometric analysis. *Agronomy*
**2023**, *13*, 1965.14.Singh, A.K.; Liu, W.; Zakari, S.; Wu, J.; Yang, B.; Jiang, X.J.; Zhu, X.; Zou, X.; Zhang, W.; Chen, C.; et al. A global review of rubber plantations: Impacts on ecosystem functions, mitigations, future directions, and policies for sustainable cultivation. *Sci. Total Environ.*
**2021**, *796*, 148948.15.Manivong, V.; Cramb, R.A. Economics of smallholder rubber expansion in Northern Laos. *Agroforest Syst.*
**2008**, *13*, 512–526.16.Weerathamrongsak, P.; Wongsurawat, W. The rubber industry of Thailand: A review of past achievements and future prospects. *J. Agribus. Dev. Emerg. Econ.*
**2013**, *3*, 49–63.17.Sdoodee, A.; Rongsawat, S. Impact of climate change on smallholders’ rubber production in Songkhla Province, Southern Thailand. In Proceedings of the 2012 International and National Conference for The Sustainable Community Development of “Local Community: The Foundation of Development in the ASEAN Economic Community (AEC)”, Kosa Hotel, Khon Kaen, Thailand, 16–19 February 2012; pp. 81–86.18.Touch, V.; Tan, D.K.; Cook, B.R.; Li Liu, D.; Cross, R.; Tran, T.A.; Utomo, A.; Yous, S.; Grunbuhel, C.; Cowie, A. Smallholder farmers’ challenges and opportunities: Implications for agricultural production, environment and food security. *J. Environ. Manag.*
**2024**, *370*, 122536.39.Johanson, D.; Wyse, D.; Janes, K. Controlling weeds phytopathogenic bacteria. *Weed Tech.*
**1996**, *10*, 621–624.41.Hadi Ismail, M.Z.; Mahyudin, M.M.; Noran, A.S.; Zambri, A.M.A.; Maiden, N.A.; Atan, S.; Aris, M.N.M. Identification and characterisation of causal pathogens of *Pestalotiopsis* leaf fall disease in *Hevea brasiliensis* using a detached leaf technique. *J. Rubber Res.*
**2024**, *27*, 159–173.42.Ma, X.; Qin, Y.; Xiang, Y.; He, L.; Song, F.; Wang, Z.; Jiang, Y.; Wu, L. First report of *Neopestalotiopsis rosae* causing leaf blight on Shatangju in southern China. *Plant Dis.*
**2023**, *107*, 2535.

The authors state that the scientific conclusions are unaffected. This correction was approved by the Academic Editor. The original publication has also been updated.
