# Supplementary material for: Fungal-Infected Weeds: A Potential Source of Leaf Spot Disease in Rubber Trees from Southern Thailand
Source: J Fungi (Basel). 2025 Mar 14;11(3):220. doi: 10.3390/jof11030220 (PMC11943287; doi:10.3390/jof11030220)
Supplement: Supplementary file 1 [file jof-11-00220-s001.zip › jof-3479594-supplementary.pdf]

Table S1. Collection details and GenBank accession numbers of *Colletotrichum* isolates in this study

Species	Culture accession No.	Host/Substrate	Location	GenBank accession			
				ITS	*GAPDH*	*ACT*	*TUB2*
C. aenigma	ICMP 18608*	*Persea americana*	Israel	JX010244	JX010044	JX009443	JX010389
	ICMP 18686	*Pyrus pyrifolia*	Japan	JX010243	JX009913	JX009519	JX010390
C. aeschynomenes	ICMP 17673 = ATCC 201874	*Aeschynomene virginica*	USA	JX010176	JX009930	JX009483	JX010392
C. alienum	ICMP 12071*	*Malus domestica*	New Zealand	JX010251	JX010028	JX009572	JX010411
	ICMP 18621	*Persea americana*	New Zealand	JX010246	JX009959	JX009552	JX010386
C. asianum	ICMP 18648 = CBS 124960	*Mangifera indica*	Panama	JX010193	JX010017	JX009546	-
	ICMP:18580 = CBS 130418*	*Coffea arabica*	Thailand	JX010196	JX010053	JX009584	JX010406
C. australianum	VPRI 43075*	*Citrus sinensis*	Australia	MG572138	MG572127	MN442109	MG572149
C. artocarpicola	MFLUCC 18-1167*	*Artocarpus heterophyllus*	Thailand	MN415991	MN435568	MN435570	MN435567
C. chrysophilum	CMM 4268*	*Musa* sp.	Brazil	KX094252	KX094183	KX093982	KX094285
C. conoides	CGMCC 3.17615	Chili pepper	China	KP890168	KP890162	KP890144	KP890174
C. endophyticum	LC 0324 = MFLU13-0418*	*Pennisetum purpureum*	Thailand	KC633854	KC832854	KF306258	MZ673954
C. fructicola	ICMP 18581 = CBS 130416	*Coffea arabica*	Thailand	JX010165	JX010033	JX009501	JX010405
	ICMP 18645 = CBS 125395	*Theobroma cacao*	Panama	JX010172	JX009992	JX009543	JX010408
C. fructicola (syn. *C. ignotum*)	ICMP 18646 = CBS 125397	*Tetragastris panamensis*	Panama	JX010173	JX010032	JX009581	JX010409
C. gloeosporioides	IMI 356878 = CBS 112999 = ICMP 17821*	*Citrus sinensis*	Italy	JX010152	JX010056	JX009531	JX010445
	CBS 119204 = ICMP 18678	*Pueraria montana* var. *lobata*	USA	JX010150	JX010013	JX009502	-
(syn. *Gloeosporium pedemontanum*)	CBS 273.51(*) = ICMP 19121	*Citrus limon*	Italy	JX010148	JX010054	JX009558	-
C. hebeiense	MFLUCC 13-0726*	*Vitis vinifera*	China	KF156863	KF377495	KF377532	KF288975
C. hystricis	CBS 142411*	*Citrus hystrix*	Italy, Catania	KY856450	KY856274	KY856023	KY856532
C. makassarense	CBS 143664*	*Capsicum annuum*	Indonesia	MH728812	MH728820	MH781480	MH846563
C. mengyinense	SAUCC0702*	*Rosa chinensis*	China	MW786742	MW846240	MW883695	MW888970
C. musae	CBS 116870	*Musa* sp.	USA	JX010146	JX010050	JX009433	JX010413
	ICMP 17817 = IMI 52264	*Musa* sp.	Kenya	JX010142	JX010015	JX009432	JX010395
C. nupharicola	CBS 469.96 = ICMP 17938	*Nuphar luteum* subsp. *polysepalum*	USA	JX010189	JX009936	JX009486	JX010397
	CBS 470.96* = ICMP 18187	*Nuphar luteum* subsp. *polysepalum*	USA	JX010187	JX009972	JX009437	JX010398
C. perseae	CBS 141365*	*Avocado*	Israel	KX620308	KX620242	KX620145	KX620341
C. queenslandicum	ICMP 1778*	*Carica papaya*	Australia	JX010276	JX009934	JX009447	JX010414
	ICMP 18705	*Coffea* sp.	Fiji	JX010185	JX010036	JX009490	JX010412
C. salsolae	ICMP 19051*	*Salsola tragus*	Hungary	JX010242	JX009916	JX009562	JX010403
	CBS 119296	*Glycine max* (innoculated)	Hungary	JX010241	JX009917	JX009559	-
C. siamense	ICMP 18578* = CBS 130417	*Coffea arabica*	Thailand	JX010171	JX009924	JX009518	JX010404

Species	Culture accession No.	Host/Substrate	Location	GenBank accession			
				ITS	*GAPDH*	*ACT*	*TUB2*
	ICMP 12567	*Persea americana*	Australia	JX010250	JX009940	JX009541	JX010387
	ICMP 17795	*Malus domestica*	USA	JX010162	JX010051	JX009506	JX010393
	1 SP12-1	*Hevea brasiliensis*	Thailand	LC746228	LC746231	LC746234	LC746240
(syn. *C. hymenocallidis*)	CBS 125378	*Hymenocallis americana*	China	JX010278	JX010019	JX009441	JX010404
(syn. *C. pandanicola*)	MFLUCC 17-0571	*Pandanaceae*	Thailand	MG646967	MG646934	MG646938	MG646926
C. tainanense	CBS 143666*	*Capsicum annuum*	Taiwan	MH728818	MH728823	MH781475	MH846558
C. tropicale	CBS 124949* = ICMP 18653	*Theobroma cacao*	Panama	JX010264	JX010007	JX009489	JX010407
	CBS 124943	*Annona muricata*	Panama	KP703360	JX010014	JX009570	KP703431
C. viniferum	GZAAS 5.08601*	*Vitis vinifera* cv. *Shuijing*	China	JN412804	JN412798	JN412795	N/A
C. xishuangbannaense	MFLUCC 19-0107*	*Magnolia liliifera*	China	MW346469	MW537586	MW652294	N/A
C. curcumae	IMI 288937	*Curcuma longa*	India	GU227893	GU228285	GU227991	GU228187
C. truncatum	CBS 151.35	*Arachis hypogaea*	Australia, Bangladesh	GU227862	GU228254	GU227960	GU228156

* = ex-holotype or authentic culture. The isolates from this research are marked in bold.

Table S2. Collection details and GenBank accession numbers of *Lasiodiplodia* isolates in this study.

Species	Isolate	Location	Substrate	GenBank Accession number		
				ITS	*TEF1α*	*TUB2*
Diplodia mutila	CMW 7060	Netherlands	*Fraxinus excelsior*	AY236955	AY236904	AY236933
D. seriata	CBS 112555[T]	Portugal	*Vitis vinifera*	AY259094	AY573220	DQ458856
Lasiodiplodia acaciae	CBS 136434[T]	Indonesia	*Acacia* sp.	MT587421	MT592133	MT592613
L. avicenniae	CMW 41467[T]	South Africa	*Avicennia marina*	KP860835	KP860680	KP860758
	LAS 199	South Africa	*Avicennia marina*	KU587957	KU587947	KU587868
L. avicenniarum	MFLUCC 17-2591[T]	Thailand	*Avicennia marina*	MK347777	MK340867	N/A
L. brasiliensis	CMM 4015[T]	Brazil	*Mangifera indica*	JX464063	JX464049	N/A
	CMM 4469	Brazil	*Anacardium occidentale*	KT325574	KT325580	N/A
	CMM 2321	Brazil	*Carica papaya*	KY783475	KY848612	KY848556
	CMW 35884	South Africa	*Adansonia* sp.	KU887094	KU886972	KU887466
L. bruguierae	CMW 41470[T]	South Africa	*Bruguiera gymnorrhiza*	KP860832	KP860677	KP860755

	CMW 42480	South Africa	*Bruguiera gymnorrhiza*	KP860834	KP860679	KP860757
L. chiangraiensis	MFLUCC 21-0003[T]	Thailand	Unknown host	MW760854	MW815630	MW815628
	GZCC 21-0003[T]	Thailand	Unknown host	MW760853	MW815629	MW815627
L. chonburiensis	MFLUCC 16-0376[T]	Thailand	*Pandanus* sp.	MH275066	MH412773	MH412742
L. gilanensis	CBS 124704[T]	Iran	*Citrus* sp.	GU945351	GU945342	KU887511
	CBS 124705	Iran	*Citrus* sp.	GU945352	GU945341	KU887510
(*L. missouriana*)	CBS 128311	USA	*Vitis vinifera*	HQ288225	HQ288267	HQ288304
L. iranensis	CBS 124710[T]	Iran	*Salvadora persica*	GU945348	GU945336	KU887516
	CBS 124711	Iran	*Juglans* sp.	GU945347	GU945335	KU887517
(*L. jatrophicola*)	CMM 3610	Brazil	*Jatropha curcas*	KF234544	KF226690	KF254927
L. krabiensis	MFLUCC 17-2617[T]	Thailand	*Bruguiera* sp.	MN047093	MN077070	N/A
L. lignicola	CBS 134112[T]	Thailand	Dead wood	JX646797	KU887003	JX646845
	CGMCC 3.18061	China	Woody branch	KX499889	KX499927	KX500002
L. mahajangana	CMW 27801[T]	Madagascar	*Terminalia catappa*	FJ900595	FJ900641	FJ900630
	CMW 27818	Madagascar	*Terminalia catappa*	FJ900596	FJ900642	FJ900631
(*L. caatinguensis*)	CMM 1325	Brazil	*Citrus sinensis* Brazil	KT154760	KT008006	KT154767
(*L. exigua*)	CBS 137785	Tunisia	*Quercus ilex*	KJ638317	KJ638336	KU887509
L. microcondia	CGMCC 3.18485[T]	Laos	*Aquilaria crassna*	KY783441	KY848614	N/A
L. plurivora	STE-U 5803[T]	South Africa	*Prunus salicina*	EF445362	EF445395	KP872421
	STE-U 4583	South Africa	*Vitis vinifera*	AY343482	EF445396	KU887525
L. pontae	CMM 1277[T]	Brazil	*Spondias purpurea*	KT151794	KT151791	KT151797
L. pseudotheobromae	CBS 116459[T]	Costa Rica	*Gmelina arborea*	EF622077	EF622057	EU673111
	CBS 116460	Costa Rica	*Acacia mangium*	EF622078	EF622058	KU198428
L. thailandica	CBS 138760[T]	Thailand	*Mangifera indica*	KJ193637	KJ193681	N/A
	CBS 138653	Thailand	*Phyllanthus acidus*	KM006433	KM006464	N/A
(*L. hyalina*)	CGMCC 3.17975	China	*Acacia confusa*	KX499879	KX499917	KX499992
(*L. swieteniae*)	MFLUCC 18-0244	Thailand	*Swietenia mahagoni*	MK347789	MK340870	MK412877
L. theobromae	CBS 164.96[T]	Papua	Fruit along coral reef coast	AY640255	AY640258	KU887532

	CBS 111530	USA	*Leucospermum* sp.	EF622074	EF622054	KU887531
L. tropica	CGMCC 3.18477[T]	Laos	*Aquilaria crassna*	KY783454	KY848616	KY848540
L. viticola	CBS 128313[T]	USA	*Vitis vinifera*	HQ288227	HQ288269	HQ288306
	UCD 2604MO	USA	*Vitis vinifera*	HQ288228	HQ288270	HQ288307

T=Ex-type strain

Table S3. Collection details and GenBank accession numbers of *Neopestalotiopsis* isolates in this study.

Species	Culture accession No.	Host/Substrate	Location	GenBank accession		
				**ITS**	***TUB2***	***TEF1***
Neopestalotiopsis alpapicalis	MFLUCC 17-2544^{T}	*Rhizophora mucronata*	Thailand	MK357772	MK463545	MK463547
N. acrostichi	MFLUCC 17-1754^{T}	*Acrostichum aureum*	Thailand	MK764272	MK764338	MK764316
	MFLUCC 17-1755	*Acrostichum aureum*	Thailand	MK764273	MK764339	MK764317
N. aotearoa	CBS 367.54; ATCC 11763; QM 381*	Canvas	New Zealand	KM199369	KM199454	KM199526
N. asiatica	MFLUCC 12-0286; NN0476380*	Unidentified tree	China	JX398983	JX399018	JX399049
N. australis	CBS 114159; STE-U 3017*	*Telopea* sp.	Australia: New South Wales	KM199348	KM199432	KM199537
	KNU16-005	Soil	Brazil	KY549598	KY549633	KY549595
N. brachiata	MFLUCC 17-1555T	*Rhizophora apiculata*	Thailand	MK764274	MK764340	MK764318
N. brasiliensis	COAD 2166T	*Psidium guajava*	Brazil	MG686469	MG692400	MG692402
	ZY4-2D	*Castanea mollissima*	China, Sichuan Province	MW166230	MW218523	MW199749
N. chrysea	MFLUCC 12-0261; NN042855*	Dead leaves	China	JX398985	JX399020	JX399051
	MFLUCC 12-0262	Dead plant	China	JX398986	JX399021	JX399052
N. clavispora	CBS 447.73	Decaying wood	Sri Lanka	KM199374	KM199443	KM199539
	MFLUCC 12-0281; NN043133*	*Magnolia* sp.	China	JX398979	JX399014	JX399045
N. coffeae- arabicae	HGUP 4015	*Coffea arabica*	China	KF412647	KF412641	KF412644
	HGUP 4019^{T}	*Coffea arabica*	China	KF412649	KF412643	KF412646
N. cubana	CBS 600.96; INIFAT C96/44-4*	Leaf litter	Cuba	KM199347	KM199438	KM199521
	PSU-R-L01	*Hevea brasiliensis*	Thailand: Narathiwat	LC521860	LC521874	LC521868
	PSU-W-L01	*Hevea brasiliensis*	Thailand: Narathiwat	LC521857	LC521878	LC521872
N. dendrobii	MFLUCC 14-0106T	*Dendrobium cariniferum*	Chiang Rai, Thailand	MK993571	MK975835	MK975829
	MFLUCC 14-0099	*Dendrobium cariniferum*	Chiang Rai, Thailand	MK993570	MK975834	MK975828
N. egyptiaca	CBS 140162T	*Mangifera indica*	Egypt	KP943747	KP943746	KP943748
N. ellipsospora	CBS 115113; HKUCC 9136	*Ardisia crenata*	Hong Kong	KM199343	KM199450	KM199544
	MFLUCC 12-0283*	Dead plant materials	China	JX398980	JX399016	JX399047
N. eucalypticola	CBS 264.37; BBA 5300*	*Eucalyptus globulus*	–	KM199376	KM199431	KM199551
N. eucalyptorum	CBS 147684T	*Eucalyptus globulus*	Fundão, Portugal	MW794108	MW802841	MW805397
	MEAN 1323	*Eucalyptus globulus*	Pegões, Portugal	MW794099	MW802832	MW805412
N. foedans	CGMCC 3.9123*	Mangrove plant	China	JX398987	JX399022	JX399053
	CGMCC 3.9202	Calliandra haematocephala	China	JX398988	JX399023	JX399054
N. formicarum	CBS 115.83	Plant debris	Cuba	KM199344	KM199444	KM199519
	CBS 362.72*	Dead Formicidae (ant)	Ghana	KM199358	KM199455	KM199517
	PSU-R-L02	*Hevea brasiliensis*	Thailand: Narathiwat	LC521861	LC521875	LC521869
N. hadrolaeliae	COAD 2637T	*Hadrolaelia jongheana*	Minas Gerais, Brazil	MK454709	MK465120	MK465122
N. hispanica	CBS 147686T	*Eucalyptus globulus*	Fundão, Portugal	MW794107	MW802840	MW805399
	CBS 147687	*Eucalyptus globulus*	Spain	MW794113	MW802846	MW805401
N. honoluluana	CBS 111535; STE-U 2078	*Telopea* sp.	USA: Hawaii	KM199363	KM199461	KM199546
N. hydeana	MFLUCC 20-0132	*Artocarpus heterophyllus*	Thailand	MW266069	MW251119	MW251129
	MFLUCC 20-0129	*Garcinia mangostana*	Thailand	MW266072	MW251122	MW251132
N. iberica	CBS 147688T	*Eucalyptus globulus*	Pegões, Portugal	MW794111	MW802844	MW805402
	CBS 147689	*Eucalyptus globulus*	Spain	MW794114	MW802847	MW805403
N. iranensis	CBS 137768T	*Fragaria × ananassa*	Iran	KM074048	KM074057	KM074051
N. javaensis	CBS 257.31*	*Cocos nucifera*	Indonesia: Java	KM199357	KM199437	KM199543
N. macadamiae	BRIP 63737cT	*Macadamia integrifolia*	New South Wales, Australia	KX186604	KX186654	KX186627

	BRIP 63737b	*Macadamia integrifolia*	New South Wales, Australia	KX186600	KX186653	KX186626
N. maddoxii	BRIP 72260a	*Macadamia integrifolia*	Australia	MZ303780	MZ312673	MZ344165
	BRIP 72266a^{T}	*Macadamia integrifolia*	Australia	MZ303782	MZ312675	MZ344167
N. magna	MFLUCC 12-0652T	*Pteridium* sp.	France	KF582795	KF582793	KF582791
N. mesopotamica	CBS 336.86*	*Pinus brutia*	Iraq	KM199362	KM199441	KM199555
	CBS 299.74	*Eucalyptus* sp.	Turkey	KM199361	KM199435	KM199541
N. musae	MFLUCC 15-0776T	*Musa* sp.	Thailand	NR_156311	KX789686	KX789685
N. natalensis	CBS 138.41T	*Acacia mollissima*	South Africa	NR_156288	KM199466	KM199552
N. nebuloides	BRIP 66617	*Sporobolus jacquemontii*	Australia, Queensland, Eton	MK966338	MK977632	MK977633
N. olumideae	BRIP 72273a^{T}	*Macadamia integrifolia*	Australia	MZ303790	MZ312683	MZ344175
	BRIP 72283a	*Macadamia integrifolia*	Australia	MZ303791	MZ312684	MZ344176
N. petila	MFLUCC 17-1738T	*Rhizophora apiculata*	Thailand	MK764276	MK764342	MK764320
N. phangngaensis	MFLUCC 18-0119T	*Pandanus* sp.	Thailand	MH388354	MH412721	MH388390
N. piceana	CBS 394.48*	*Picea* sp.	UK	KM199368	KM199453	KM199527
N. protearum	CBS 114178; STE-U 1765*	*Leucospermum cuneiforme* cv. *'Sunbird'*	Zimbabwe	JN712498	KM199463	KM199542
N. rhizophorae	MFLUCC 17-1551T	*Rhizophora mucronata*	Thailand	MK764277	MK764343	MK764321
N. rosae	CBS 101057*	*Rosa* sp.	New Zealand	KM199359	KM199429	KM199523
N. rosicola	CFCC 51992T	*Rosa chinensis*	China	KY885239	KY885245	KY885243
N. samarangensis	MFLUCC 12-0233T	*Syzygium samarangense*	Thailand	JQ968609	JQ968610	JQ968611
N. saprophytica	CBS 115452; HKUCC 8684	*Litsea rotundifolia*	Hong Kong	KM199345	KM199433	KM199538
	MFLUCC 12-0282T	*Magnolia* sp.	China	JX398982	JX399017	JX399048
N. sichuanensis	CFCC 54338	*Castanea mollissima*	China, Sichuan Province	MW166231	MW218524	MW199750
	SM15-1C	*Castanea mollissima*	China, Sichuan Province	MW166232	MW218525	MW199751
N. sonneratae	MFLUCC 17-1745T	*Sonneronata alba*	Thailand	MK764280	MK764346	MK764324
N. steyaertii	IMI 192475T	*Eucalyptus viminalis*	Australia	KF582796	KF582794	KF582792
N. surinamensis	CBS 450.74*	Soil under *Elaeis guineensis*	Suriname	KM199351	KM199465	KM199518
	CBS 111494	*Protea eximia*	Zimbabwe	JX556232	KM199462	KM199530
N. thailandica	MFLUCC 17-1730T	*Rhizophora mucronata*	Thailand	MK764281	MK764347	MK764325
	MFLUCC 17-1731	*Rhizophora mucronata*	Thailand	MK764282	MK764348	MK764326
N. umbrinospora	MFLUCC 12-0285T	unidentified plant	China	JX398984	JX399019	JX399050
N. vheenae	BRIP 72293a^{T}	*Macadamia integrifolia*	Australia	MZ303792	MZ312685	MZ344177
	BRIP 70210	*Macadamia integrifolia*	Australia	MN114212	MN114214	MN114213
N. vitis	MFLUCC 15-1265T	*Vitis vinifera*	China	KU140694	KU140685	KU140676
	MFLUCC 15-1266	*Vitis vinifera*	China	KU140695	KU140686	KU140677
N. zakeelii	BRIP 72271a	*Macadamia integrifolia*	Australia	MZ303788	MZ312681	MZ344173
	BRIP 72282a^{T}	*Macadamia integrifolia*	Australia	MZ303789	MZ312682	MZ344174
N. zimbabwana	CBS 111495; STE-U 1777*	*Leucospermum cunciforme* cv. *'Sunbird'*	Zimbabwe	JX556231	KM199456	KM199545
Pestalotiopsis trachicarpicola	MFLUCC 12-0263; NN0470720	Unidentified tree	China	JX399000	JX399031	JX399064
Pestalotiopsis trachicarpicola	OP068; IFRDCC 2440*	*Trachycarpus fortunei*	China	JQ845947	JQ845945	JQ845946

* = ex-holotype or ex-epitype culture. The isolates from this research are marked in bold.
